# Composition and Functional Diversity of Epiphytic Bacterial and Fungal Communities on Marine Macrophytes in an Intertidal Zone

**DOI:** 10.3389/fmicb.2022.839465

**Published:** 2022-03-18

**Authors:** Jun Chen, Yu Zang, Zhibo Yang, Tongfei Qu, Tao Sun, Shuo Liang, Meiling Zhu, Ying Wang, Xuexi Tang

**Affiliations:** ^1^College of Marine Life Sciences, Ocean University of China, Qingdao, China; ^2^Key Laboratory of Marine Eco-Environmental Science and Technology, First Institute of Oceanography, Ministry of Natural Resources, Qingdao, China; ^3^Laboratory for Marine Ecology and Environmental Science, Qingdao National Laboratory for Marine Science and Technology, Qingdao, China

**Keywords:** epiphytic microbial community, 16SrRNA, ITS, macroalgae, seagrass

## Abstract

Marine macrophytes (seagrasses and macroalgae) and their epiphytic microorganisms play an important role in the ecological and biochemical processes of coastal oceans. However, simultaneous comparative studies on the biodiversity and functions of epiphytic bacteria and fungi associated with marine macrophytes have not been conducted. In this study, high-throughput sequencing technology was used to describe the epiphytic bacterial and fungal communities of 11 common macroalgae and 2 seagrasses from an intertidal zone of northern China and compare them with seawater communities. The results showed that Proteobacteria and Bacteroidota were the dominant bacterial phyla in marine macrophytes, whereas Ascomycota, Chytridiomycota, and Basidiomycota were the dominant fungal phyla. The alpha diversity of the bacterial and fungal communities in seagrasses was the highest of all macrophyte samples. This may have been related to their ability to recruit microorganisms from multiple sources. Host phylogeny may influence bacterial community structure, and geographical differences may influence fungal community structure. The FAPROTAX data indicated that C metabolic microbes were enriched in marine macrophytes, while the FUNGuild data indicated that undefined saprotroph, which participated in organic matter degradation, were also enriched in marine macrophytes. These findings provide a theoretical basis regarding the epiphytic microorganisms of macrophytes and may offer new insights to support the improved ecological restoration of seagrass and macroalgae beds.

## Introduction

As primary producers, seagrass and macroalgae beds are critical marine ecosystems. They serve a variety of ecosystem functions, including providing food for marine herbivores (Alcoverro and Mariani, 2004; Shpigel et al., 2017), acting as nurseries (Verweij et al., 2008), providing coastal protection (Christianen et al., 2013), purifying seawater bodies, and mitigating climate change through blue carbon sequestration (Duarte et al., 2018). Seagrass and green, brown, and red macroalgae are currently receiving increasing research attention related to the development of a more sustainable (blue) economy (Choudhary et al., 2021). Despite their acknowledged importance, seagrass and macroalgae beds are showing signs of degradation in many regions of the world under the influence of various factors such as global climate change and human interference (Zhang and Sun, 2007; Dunic et al., 2021). Therefore, the protection of marine macrophytes (seagrasses and macroalgae) has become a focus of environmental protection throughout the world, and research on these macrophytes has become a frontier of coastal ecological research.

Marine macrophytes play an important role in the ecological and biochemical processes of the coastal ocean, forming close associations with microorganisms belonging to all three domains of life (Egan et al., 2013; Tarquinio et al., 2019; Korlević et al., 2021). Microbes can live on the surface of macrophytes in very active association with their hosts (Wahl et al., 2012). These microbes (bacteria and fungi) provide hormones, vitamins, minerals, carbon dioxide, and numerous novel bioactive metabolites to macrophytes, thus playing important roles in marine macrophyte morphogenesis, growth, and immune defense. In turn, marine macrophytes provide habitats, oxygen, and carbohydrates such as algal polysaccharides to associated microbes (Cho et al., 2015; Kouzuma and Watanabe, 2015; Singh et al., 2015; Mei et al., 2019). Phyllosphere microbiota associated with terrestrial plants and their interactions with plant hosts have been intensively investigated (Gong and Xin, 2021). However, fewer studies have simultaneously researched the epiphytic bacteria and fungi of marine macrophytes.

The community structure of epiphytic microorganisms associated with macroalgae and seagrasses is host-specific and differs among different macroalgae and seagrasses. For example, a study on the culturable epibiotic fungi of seaweeds in the Red Sea, Egypt, showed that seaweed hosts showed a strong selective pressure on the epiphytic fungal community and thus could control temporal variation in the fungal assemblage (Abdel-Gawad et al., 2014). Furthermore, a study on the structure of epiphytic bacterial communities of eight common macroalgae growing in the intertidal zone of Cape Vidal, South Africa showed that the bacterial communities of brown macroalgae were very similar to those of green macroalgae, while those of red macroalgae were different. This distinction was not due to the host phylogeny but was better explained by algal organic exudates as well as elemental deposits on the hosts’ surfaces, instead (Selvarajan et al., 2019). Similarly, a study on the function and diversity of microbial communities on different types of host surfaces (e.g., macroalgae and seagrasses) found that taxonomic diversity was unique to each type of host and was best explained by the host’s physicochemical properties (Roth-Schulze et al., 2016). Metagenomic analysis has shown that despite differences in microbial community composition among host species, most of the microbial functions are conserved, suggesting that the functions of different epiphytic microbial species may be similar (Burke et al., 2011; Roth-Schulze et al., 2016; Cucio et al., 2018). This observation can be explained by the Lottery Hypothesis (Sale, 1976), which assumes that an initial random colonization step takes place from a set of functionally equivalent taxonomic groups, resulting in taxonomically different epiphytic communities sharing a core set of functional genes (Burke et al., 2011; Schmidt et al., 2015; Roth-Schulze et al., 2016).

In addition, some studies have also shown that seasonal variations, spatial differences, and environmental factors can also have an effect on the surface epiphytic microbial composition of macroalgae and seagrasses (Lachnit et al., 2011; El-Said and El-Sikaily, 2013; Korlević et al., 2021). To exclude the effects of the above factors as much as possible, the present study explored the differences between the epiphytic microorganisms of marine macrophytes from different hosts. In this study, a region rich in macroalgae and seagrass was selected. The dominant species in this region were selected, and the diversity of epiphytic communities of 13 different macroalgae and seagrass was investigated. In addition, this study analyzed the microbial characteristics of the seawater communities, compared them with those on living surfaces (seagrasses and macroalgae), and predicted their functional differences. To our knowledge, this is the first study in China to investigate epiphytic microbial communities on different species of macroalgae and seagrass using next-generation sequencing technology.

## Materials and Methods

### Sample Collection and Microbial Enrichment

**FIGURE 1:**
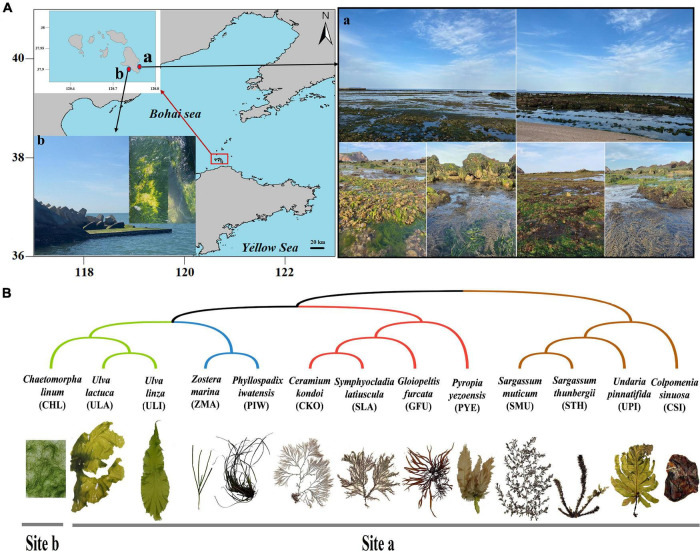
Sample Information. **(A)** Sampling locations. **(B)** Phylogenetic relationships and collection locations of different marine macrophytes.

Fourteen different types of samples were collected from the intertidal zones of Changdao County, Yantai City, China, during the afternoon on April 13, 2021, and identified by their morphological features (Figure 1). This area had a highly diverse community of macroalgae and seagrasses. The 14 types of samples included 3 types of green macroalgae (*Ulva linza*, *Ulva lactuca*, and *Chaetomorpha linum*), 4 types of red macroalgae (*Gloiopeltis furcata*, *Symphyocladia latiuscula*, *Ceramium kondoi*, and *Pyropia yezoensis*), 4 types of brown macroalgae (*Undaria pinnatifida*, *Colpomenia sinuosa*, *Sargassum thunbergii*, and *Sargassum muticum*), 2 types of seagrasses (*Zostera marina* and *Phyllospadix iwatensis*), and 1 seawater sample (Figure 1B). All samples were collected during low tide in a rectangular area of about 200 m × 30 m, except for *C. linum*, which was collected 5 km away. Among them, except for *U. lactuca*, *Z. marina*, and *P. iwatensis* with two biological replicates, the other samples were all three biological replicates. Macroalgae attached to rocky surfaces and seagrasses growing in the sediment were collected using seawater-washed forceps and needles. Seawater samples were collected in 1-L sterile plastic bottles. All of the samples were placed in sterile plastic, stored in a portable icebox, and then transferred to the laboratory for further analysis. Each sample was rinsed with sterile artificial seawater to remove loosely attached epibionts and sand. Approximately 25 g of each sample was placed in a conical flask containing 70 mL phosphate-buffered saline (PBS) buffer (1 mmol/L) and then placed in an oscillating incubator shaking the flask at 200 R.min^–1^ for 30 min. The eluent was filtered through a sterile (10 cm × 10 cm) 0.22-μm gauze membrane. Similarly, the seawater samples were filtered through a 0.22-μm filter membrane directly after the sterile silk sieve. After microbial enrichment, the filter membrane was stored at –80°C and sent to the sequencing company on dry ice.

### DNA Extraction, 16S rRNA and ITS Gene Polymerase Chain Reaction, and Sequencing

Total genomic DNA was isolated from each sample using a FastDNA™ SPIN Kit (MP Biomedicals, Solon, United States) according to the manufacturer’s recommended protocol. The DNA extract was checked on 1% agarose gel, and the DNA concentration and purity were determined with a NanoDrop 2000 UV-vis spectrophotometer (Thermo Fisher Scientific, Wilmington, United States). The V3–V4 region of 16S rRNA genes and the fungal ITS1 region were amplified using the primer pairs 338F and 806R (forward primer, 5′-ACTCCTACGGGAGGCAGCAG-3′; reverse primer 5′-GGACTACHVGGGTWTCTAAT-3′) and ITS1F and ITS2R (forward primer, 5′-CTTGGTCATTTAGAGGAAGTAA-3′; reverse primer 5′-GCTGCGTTCTTCATCGATGC-3′) by an ABI GeneAmp^®^ 9700 PCR thermocycler (ABI, CA, United States). The following Polymerase Chain Reaction (PCR) protocol was used: 3 min at 95°C for initial denaturation, 35 (ITS) and 29 (16S) cycles of 30 s at 95°C for denaturation, 30 s at 55°C for annealing, 30 s at 72°C for elongation, and a final extension at 72°C for 10 min. All PCR products were extracted with 2% agarose gel and purified using the AxyPrep DNA Gel Extraction Kit (Axygen Biosciences, Union City, CA, United States) according to manufacturer’s instructions and quantified using Quantus™ Fluorometer (Promega, United States). High-throughput sequencing of bacterial rRNA genes and fungal ITS genes was performed by Majorbio Bio-Pharm Technology Co., Ltd. (Shanghai, China) using an Illumina MiSeq PE300 platform (Illumina, San Diego, United States) according to the standard protocols. Sequences obtained in this study have been deposited in the National Center for Biotechnology Information (NCBI) under the accession number PRJNA805355.

### Bioinformatics Analysis

The raw 16S rRNA and ITS gene sequencing reads were demultiplexed, quality-filtered using fastp version 0.20.0 (Chen et al., 2018), and merged by FLASH version 1.2.7 (Magoč and Salzberg, 2011) with the following criteria: (i) the reads were truncated at any site with an average quality score < 20 over a 50-bp sliding window; (ii) the primers were exactly matched allowing two-nucleotide mismatches, and reads containing ambiguous bases were removed; and (iii) only overlapping sequences longer than 10 bp were assembled according to their overlapped sequence. Operational taxonomic units (OTUs) with a 97% similarity cut-off were clustered using UPARSE version 7.1 (Edgar, 2013), and chimeric sequences were identified and removed. The representative OTU sequences were annotated using the SILVA bacterial 16S rRNA database (Release138) (Quast et al., 2012) and the UNITE fungal ITS database (Release 8.1) (Kõljalg et al., 2013) using a QIIME-based wrapper of RDP-classifier v.2.2 with a confidence cut-off of 0.7 (Wang et al., 2007). Data normalization was based on the smallest number of effective sequences in all samples. The subsequent alpha diversity analysis and beta diversity analysis were based on the normative data. The detected communities were identified and annotated at different taxonomic levels (phylum, class, order, family, genus, and species). Further analysis was performed to calculate the alpha diversity and richness of OTUs. The community composition of each sample was determined at different classification levels. Alpha diversity was evaluated by calculating six indices, specifically the Observed-species, Chao1, Shannon, Simpson, ACE, and Good-coverage indices. All of the indices were calculated with Mothur software (version 1.30.2) (Schloss et al., 2009). Non-metric multidimensional scaling (NMDS) based on the Bray-Curtis distances was applied using R v.3.5.3 to reduce the dimensions of the original variables and statistically compared using the ANOSIM analysis for the data shown. Linear discriminant analysis (LDA) coupled with effect size measurement (LEfSe) analysis was applied using LEfSe software to search for statistically different biomarkers between different groups (Segata et al., 2011). The FAPROTAX and FUNGuild were used to infer the functional profiles of the microbiota communities (Louca et al., 2016; Nguyen et al., 2016).

## Results

### Characterization of Illumina Sequencing Data

Using the Illumina MiSeq sequencing platform, we obtained a total of 4,840,020 raw reads for 78 samples. After removing the low-quality sequences and mismatches, a total of 4,386,231 clean sequence reads were obtained. Finally, the chimeras and chloroplast and mitochondrial sequences were filtered to obtain the effective sequence reads for subsequent analysis. For bacteria, 1,120,148 effective sequence reads with bacterial species annotations were obtained from 39 samples, with an average of 28,722 effective sequence reads per sample (range: from 17,044 to 37,232). When the normalized sequences (17,044 reads) at a level of 97% similarity were grouped, they resulted in a normalized dataset comprising 5,683 OTUs. For fungi, 24,26,268 effective sequence reads were obtained, with an average of 62,121 effective sequence reads per sample (range from 31,762 to 74,536). When the read number was normalized to 31,762, it resulted in a normalized dataset comprising 3,529 fungal OTUs (Supplementary Table 1). The rarefaction curve analysis showed that the curves gradually flattened when the bacterial samples were sequenced up to 4,000 and the fungal samples were sequenced up to 5,000 (Supplementary Figure 1), indicating that the amount of sequencing data was reasonable and reflected the diversity of microorganisms in the samples.

### Venn Diagram Analysis of Operational Taxonomic Units

**FIGURE 2:**
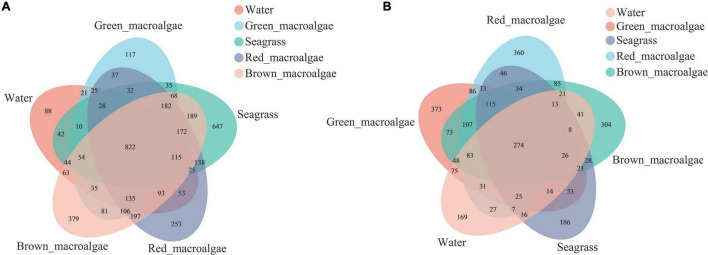
Venn diagrams showing the unique and shared operational taxonomic units (OTUs) between the seawater, green macroalgae, red macroalgae, brown macroalgae, and seagrass. **(A)** Venn diagrams of bacterial OTUs. **(B)** Venn diagrams of fungal OTUs.

A Venn diagram was used to count the number of common and unique species in multiple samples, which could visually represent the compositional similarity and overlap of samples at the OTU level. Bacterial OTU analysis showed that 822 OTUs were shared among all of the samples (Figure 2A); the number of bacterial OTUs shared between each group followed the order: brown macroalgae and red macroalgae (197) > brown macroalgae and seagrass (189) > red macroalgae and seagrass (138) > brown macroalgae and green macroalgae (81) > brown macroalgae and seawater (63). Among them, red macroalgae, brown macroalgae, and seagrass shared the highest number of bacterial OTUs (172). Fungal OTU analysis showed that 274 OTUs were shared across all samples (Figure 2B); the number of fungal OTUs shared between each group followed the order: green macroalgae and red macroalgae (86) > red macroalgae and brown macroalgae (85) > green macroalgae and seawater (75) > green macroalgae and brown macroalgae (73) > green macroalgae and seagrass (53). Among them, green macroalgae, red macroalgae, and brown macroalgae shared the highest number of fungal OTUs (107). The OTU numbers of both fungi and bacteria were higher in macroalgae and seagrasses than in the seawater, suggesting that macroalgae and seagrass may recruit more species of epiphytic microbiota from their habitat than those present in the seawater.

### Richness and Diversity Analyses of Epiphytic Bacterial and Fungal Communities

**FIGURE 3:**
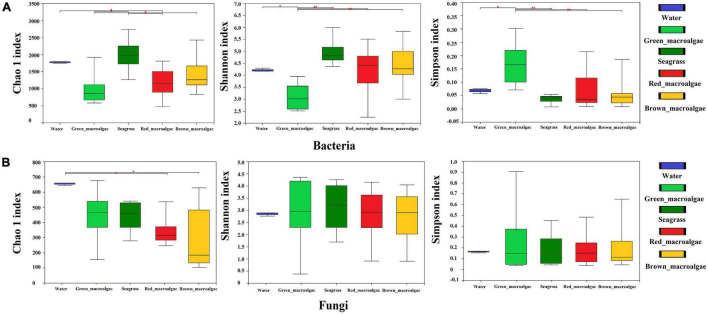
Alpha-diversity of the seawater, green macroalgae, red macroalgae, brown macroalgae, and seagrasses. **(A)** Alpha-diversity of the bacterial community. **(B)** Alpha-diversity of the fungal community. Statistical comparison of indicated data was performed using Wilcoxon rank-sum texts with * (*P* < 0.05), and ^**^ (*P* < 0.01).

The alpha diversity indices for the samples are shown in Supplementary Table 2. The Good’s coverage was between 0.95 and 0.99, indicating that the sequencing results represented the true situation of the microflora structures. The bacterial Chao1 indices of each sample followed the order: seagrass > seawater > brown macroalgae > red macroalgae > green macroalgae (Figure 3A). The fungal Chao1 indices of each sample followed the order: seawater > green macroalgae > seagrass > red macroalgae > brown macroalgae (Figure 3B). The Chao1 indices of the bacterial communities in seagrass were significantly higher than the bacterial communities in the red and green macroalgae (*p* < 0.05), and the Chao1 indices of the fungal communities in seawater were significantly higher than those of the fungal communities in red macroalgae and brown macroalgae (*p* < 0.05). This pattern indicates that seagrasses have a higher bacterial species richness than the other groups, while the seawater has a higher fungal species richness than the other groups. In addition, the bacterial communities in seagrasses had a higher Shannon index and a lower Simpson index, indicating that the species richness and evenness in seagrasses were higher than in other groups.

### Community Composition of Epiphytic Bacterial and Fungal Communities

**FIGURE 4:**
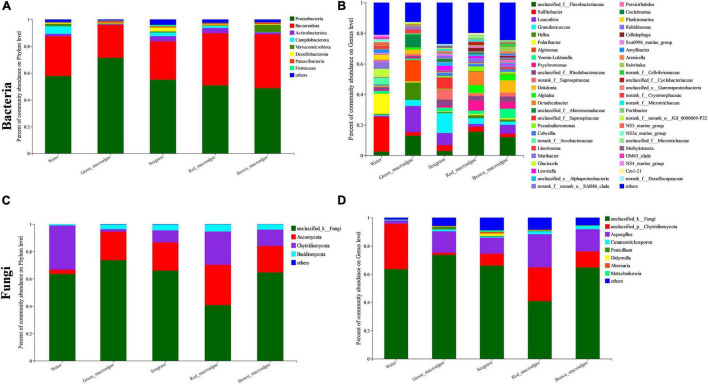
Relative abundances of bacteria and fungi in seawater, green macroalgae, red macroalgae, brown macroalgae, and seagrass. **(A)** Bacterial community composition at the phylum level. **(B)** Bacterial community composition at the genus level. **(C)** Fungal community composition at the phylum level. **(D)** Fungal community composition at the genus level.

Bacterial and fungal community structure varied among the samples. At the phylum level, Proteobacteria and Bacteroidota were the dominant groups in each sample, representing more than 71% of the total sequences in all five groups (Figure 4A and Supplementary Figure 2A). Furthermore, the relative abundances of Verrucomicrobiota in brown macroalgae were higher than in the other four groups. At the genus level, the relative abundances of bacteria varied considerably among the different groups (Figure 4B and Supplementary Figure 2B). The identified dominant genera in seawater were *Sulfitobacter* (22.97%), *Polaribacter* (13.22%), *Glaciecola* (5.20%), *Planktomarina* (3.32%), and *Amylibacter* (2.15%). The identified dominant genera in green macroalgae were *Leucothrix* (17.13%), *Algimonas* (14.06%), *Hellea* (11.36%), *Granulosicoccus* (3.97%), and *Dokdonia* (2.90%). The identified dominant genera in seagrasses were *Granulosicoccus* (12.98%), *Leucothrix* (7.97%), *Sulfitobacter* (3.86%), *Lewinella* (2.83%), and *Yoonia-Loktanella* (2.59%). The identified dominant genera in red macroalgae were *Octadecabacter* (8.73%), *Psychromonas* (6.66%), *Algitalea* (5.00%), *Granulosicoccus* (3.69%), and *Sulfitobacter* (3.27%). The identified dominant genera in brown macroalgae were *Dokdonia* (8.10%), *Yoonia-Loktanella* (6.18%), *Leucothrix* (5.72%), *Psychromonas* (4.38%), and *Algitalea* (3.74%). With respect to fungal communities, Ascomycota, Chytridiomycota, and Basidiomycota were the most abundant phyla (Figure 4C and Supplementary Figure 4C). The abundance of unclassified k fungi was highest (ranging from 40.81 to 73.59%). At the fungal genus level, the identified dominant genera were *Aspergillus*, *Cutaneotrichosporon*, *Penicillium, Didymella*, *Alternaria*, and *Metschnikowia* (Figure 4D and Supplementary Figure 2D). The fungal phylum Chytridiomycota had a higher abundance in *C. kondoi* samples than in the other samples.

### Beta Diversity Analysis of Epiphytic Bacterial and Fungal Communities

A non-parametric statistical test using “anosim” and “adonis” showed that the differences between the bacterial communities in all of the groups were greater than the differences within the groups, which indicated that the grouping was appropriate (*P* < 0.05) (Table 1). For fungal communities, the experimental results showed significant differences between most groups, except for two groups (seagrass vs. brown macroalgae and seagrass vs. brown macroalgae). The difference between seawater and the rest of the groups was greater than that between macroalgae and seagrass.

**TABLE 1:** Statistical analysis of the seawater, green macroalgae, red macroalgae, brown macroalgae, and seagrass using “adonis”.

	Diffs	Df	*F*-value	R2	*P*-value
Bacteria	Seawater vs. Green_macroalgae	1	5.526638084	0.38044853	0.005**
	Seawater vs. Seagrass	1	8.043386311	0.61666396	0.03*
	Seawater vs. Red_macroalgae	1	4.724062326	0.26653384	0.008**
	Seawater vs. Brown_macroalgae	1	5.565994857	0.29979513	0.005**
	Green_macroalgae vs. Seagrass	1	3.586472805	0.2639738	0.013*
	Green_macroalgae vs. Red_macroalgae	1	3.873676648	0.17709307	0.001**
	Green_macroalgae vs. Brown_macroalgae	1	4.586383573	0.20305967	0.001**
	Seagrass vs. Red_macroalgae	1	2.482401188	0.1506092	0.01*
	Seagrass vs. Brown_macroalgae	1	3.175321705	0.18487699	0.009**
	Red_macroalgae vs. Brown_macroalgae	1	2.858800483	0.11500155	0.001**
Fungi	Seawater vs. Green_macroalgae	1	3.95415953	0.30524246	0.011*
	Seawater vs. Seagrass	1	3.900170007	0.43821298	0.03*
	Seawater vs. Red_macroalgae	1	4.280635476	0.24771285	0.008**
	Seawater vs. Brown_macroalgae	1	2.968598197	0.18590224	0.024*
	Green_macroalgae vs. Seagrass	1	1.947163216	0.16298122	0.069
	Green_macroalgae vs. Red_macroalgae	1	2.294954672	0.11308006	0.0179*
	Green_macroalgae vs. Brown_macroalgae	1	2.967730987	0.14153801	0.012*
	Seagrass vs. Red_macroalgae	1	1.894504572	0.11919243	0.049**
	Seagrass vs. Brown_macroalgae	1	1.465477757	0.094758	0.188
	Red_macroalgae vs. Brown_macroalgae	1	3.828898154	0.14824086	0.002**

Statistical difference is designated as follows: *p < 0.05, **p < 0.01.

**FIGURE 5:**
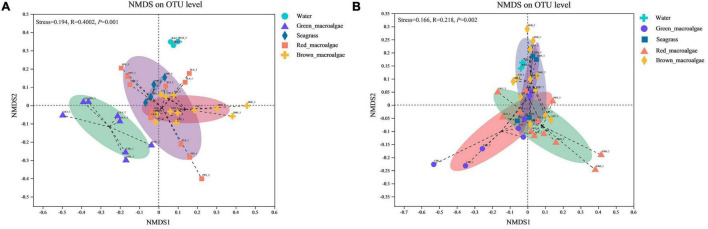
Non-metric multidimensional scaling (NMDS) ordination plot based on Bray–Curtis dissimilarity data. **(A)** NMDS of bacterial community composition. **(B)** NMDS of fungal community composition.

An NMDS ordination plot was produced based on the Bray–Curtis distance and revealed distinct differences in the structure of bacterial and fungal communities from different samples (Figure 5). For the bacterial communities, significant differences in microbial community structure were observed between different groups (Figure 5A and Table 1). The bacterial community structures of seagrass, red macroalgae, and brown macroalgae clustered together and were distinct from those of seawater and green macroalgae. For fungi, with the exception of *C. linum* (which was collected 5 km away) in the green macroalgae group, the remaining marine macrophytes clustered together (Figure 5B), indicating that samples from similar sampling locations tended to have similar fungal communities (*R*^2^ = 0.117; *p* = 0.001).

### Differential Abundance of Microbial Taxa Across Groups

**FIGURE 6:**
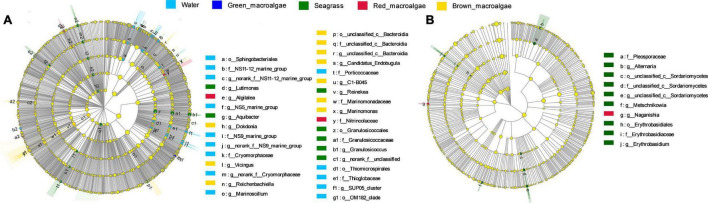
Biomarker analysis of the seawater, green macroalgae, red macroalgae, brown macroalgae, and seagrasses. Cladogram showing the phylogenetic structures of the microbiota. In the branching diagram of their evolution, the circles that radiate from inside to outside represent taxonomic levels from phylum to genus, and each small circle represents an individual taxon. The diameter of the circles is proportional to the relative abundance. Species that are not significantly different are uniformly colored yellow. **(A,B)** Refer to the bacterial samples and the fungal samples, respectively.

To explore the signature microbes among the groups from phylum to genus, LEfSe analysis was performed. For the bacterial communities, the LEfSe (LDA = 3) analysis identified 18, biomarkers for seawater, 1 for green macroalgae, 18 for seagrass, 2 for red macroalgae, and 18 for brown macroalgae (Figure 6A and Supplementary Figure 3). At the genus level, the significantly more abundant epiphytic bacteria of seagrass were the genera *Lutimonas*, *Aquibacter*, *Reinekea*, and *Granulosicoccus*; the significantly higher epiphytic bacteria of red macroalgae were in the *Algitalea* genus; the significantly more abundant epiphytic bacteria of brown macroalgae were the genera *Dokdonia*, *Vicingus*, *Reichenbachiella*, *Candidatus_Endobugula*, *C1-B045*, and *Marinomonas*; and the significantly more abundant epiphytic bacteria of seawater were the genera *NS5 marine group*, *Marinoscillum*, and *SUP05 cluster*. For fungal communities, the LEfSe (LDA = 2) analysis identified 9, biomarkers for seagrass, and 1 for red macroalgae (Figure 6B and Supplementary Figure 3). At the genus level, the genera *Alternaria*, *Metschnikowia*, and *Erythrobasidium* were significantly higher in seagrass while *Naganishia* was significantly higher in red macroalgae.

### Functional Prediction Analysis

**FIGURE 7:**
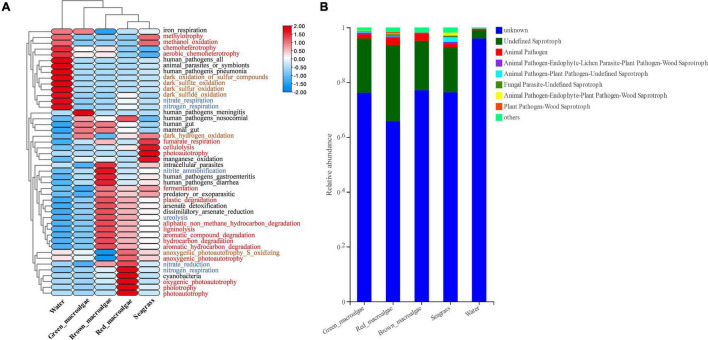
Functional prediction for epiphytic microorganisms. **(A)** Functional prediction of bacterial composition using FAPROTAX. The red, blue, and brown fonts represent C metabolism, N metabolism, and S metabolism, respectively. **(B)** Functional prediction of fungal core composition using FUNGuild.

FAPROTAX analysis was used to predict the functional capacity of the epiphytic bacteria. It was that the functions of most epiphytic bacteria were focused on carbon, nitrogen, and sulfur cycling (Figure 7A). For C metabolism, the functional enrichment abundance of macroalgae and seagrass epiphytic bacteria was higher than in seawater, except for individual functional groups such as chemoheterotrophy, methylotrophy, methanol oxidation, and anoxygenic photoautotrophy. For N metabolism, the bacterial groups associated with nitrate reduction (except for green macroalgae) and ureolysis were higher in macroalgae and seagrass groups than in seawater. While nitrate respiration and nitrogen respiration were higher in seawater than in the macroalgae and seagrass groups, the abundances of bacterial groups associated with nitrite respiration and nitrite ammonification were lower in all of the samples. In addition, the abundances of bacterial groups associated with S metabolism (such as the dark oxidation of sulfur compounds, dark sulfite oxidation, dark sulfur oxidation, and dark sulfide oxidation) and animal and human diseases (animal parasites or symbionts, human pathogens all, and human pathogens pneumonia) were significantly higher in seawater than in macroalgae and seagrasses.

Fungi were functionally classified using the FUNGuild software. Most of them could not be classified, which may have been related to the relative paucity of marine fungal databases (Figure 7B). Three ecological groups, namely undefined saprotroph, animal pathogen–endophyte–plant pathogen–wood saprotroph, and animal pathogen accounted for the top three most abundant fungal OTUs. The relative abundance of undefined saprotrophs was generally higher in the macroalgae and seagrass groups than in seawater.

## Discussion

It is well known that the surfaces of macroalgae and seagrasses harbor a variety of microbial (bacterial and fungal) symbionts that contribute to morphological development and defense mechanisms (Crump et al., 2018; Wang et al., 2018; Wichard and Beemelmanns, 2018; Califano et al., 2020). These epiphytic microbial communities are highly cosmopolitan and distinctly different from the free-living microbes in the surrounding seawater (Lachnit et al., 2011). However, to date, studies on the epiphytic bacteria and fungi of macroalgae and seagrasses have lagged behind compared to studies on terrestrial plants. Comparative studies of epiphytic bacteria and fungi in macroalgae and seagrasses have not been reported. Changdao County in Yantai, northern China, is an island at the intersection of the Yellow and Bohai Seas with an extremely rich and diverse flora of composed macroalgae and seagrasses. In this study, intertidal macroalgal and seagrass-associated epiphytic microbial communities were explored using high-throughput sequencing analysis, and the differences and functions among them were compared.

### Taxonomic Composition of Bacteria and Fungi

The phylum-level composition of epiphytic microbial communities in different macroalgal and seagrass samples was similar. However, the microbial community composition of macroalgae and seagrasses differed significantly at the genus level (Figure 4 and Supplementary Figure 2). Proteobacteria and Bacteroidetes were the two core bacterial phyla recorded in all samples. Proteobacteria has also been observed to be the most dominant in other related studies (Tujula et al., 2010; Lachnit et al., 2011). Bacteria in the Proteobacteria phylum are often ubiquitous in the environment and play an important role in promoting surface colonization and biofilm formation (Selvarajan et al., 2018). Bacteroidetes were the second-most common component in all samples, which was consistent with previous findings in many macroalgae and seagrass studies (Selvarajan et al., 2019; Garcias-Bonet et al., 2021). Bacteroidetes bacteria are well known biopolymer degraders that allow the growth of colonizing bacteria by providing an aerobic environment within the surface biofilm (Dang et al., 2011). Previous studies have reported that bacterial phyla such as Actinobacteria, Campilobacterota, Verrucomicrobia, and Firmicutes are common in many types of macroalgae (Lachnit et al., 2011; Bondoso et al., 2014; Del Olmo et al., 2018; Selvarajan et al., 2019). Consistent with the findings of this study, the relative abundances of Actinobacteria and Verrucomicrobia were more variable in different groups, suggesting that the members of these phyla play an important role in different functional traits. A large proportion (40.81–73.59%) of sequences annotated to fungi were not classified at a finer taxonomic level. According to previous studies, many plant microbiomes contain various proportions of unclassified fungi (Yao et al., 2019). This phenomenon may be due to the limited ITS gene sequence database and the scarcity of research on the classification of marine fungi. Unclassified fungi and their functions in different hosts require further research. The Ascomycota is considered one of the most widespread and diverse groups of eukaryotes. Together with the Basidiomycota, it forms the subkingdom Dikarya, which assists with the digestion of plant biopolymers such as cellulose and lignin (Hyde et al., 1998; van der Wal et al., 2013). Similar to the findings of previous studies on the diversity of culturable fungi in intertidal macroalgae (Wang et al., 2018; Ettinger et al., 2021), the present study also identified Ascomycota and Basidiomycota as the two most abundant phyla. In current studies of fungi based on sequenced environmental DNA, the Ascomycota and Basidiomycota phyla have been found to be overwhelmingly dominant in various marine environments, such as Hawaiian nearshore seawater (Gao et al., 2010), mariculture (Guo et al., 2015), nearshore marsh sediments (Mohamed and Martiny, 2011), mangrove sediments (Arfi et al., 2012), and deep-sea environments (Bernhard et al., 2014). In contrast, the present study found that Chytridiomycota was also a dominant phylum in each group. This phylum contains a large number of unknown taxa, suggesting that the ocean is the source of many species of “dark matter fungi.” Based on their study of freshwater ecosystems, Kagami et al. (2014) proposed the concept of “mycoloop” to describe the carbon and nutrient transfer pathway from algae to zooplankton via the swimming spores of parasitic chytrids. The present study also found that Chytridiomycota were dominant in marine macrophytes and seawater, suggesting that the “mycoloop” is also ecologically important in marine ecosystems.

### Changes in Bacterial and Fungal Community Diversity

Some previous studies have shown that, unlike inanimate surfaces, macroalgae and seagrass surfaces have metabolically produced substances that selectively modulate microbial adhesion and settlement from the surrounding environment to build specific microbial communities (Roth-Schulze et al., 2016; Wichard and Beemelmanns, 2018). This is often referred to as the “host effect.” Seasonal variations, spatial differences, and environmental factors can also have an effect on the composition of epiphytic microorganisms on the surface of macroalgae and seagrass (Lachnit et al., 2011; El-Said and El-Sikaily, 2013; Korlević et al., 2021). The β-diversity results in this study showed that microbial communities differed on different host species, but this host specificity did not extend to a higher taxonomic level (e.g., red macroalgae) (Figure 5). For bacterial communities, host phylogeny may have played a role in the selection of microbial communities, especially for green macroalgae (Figure 5A). Therefore, it can be speculated that other factors, such as micro-environmental (e.g., surface pH) or chemical properties of the host (e.g., specific organic carbon availability), might drive community assembly. Consistent with this hypothesis, Selvarajan et al. (2019) found that the diversity of epiphytic bacteria on macroalgae surfaces was greatly influenced by algal organic exudates as well as elemental deposits on their surfaces, which triggered chemotaxis responses from epiphytic bacteria with the genes needed to metabolize those substrates (Selvarajan et al., 2019). For fungal communities, the present study found that the difference between fungal communities was smaller than that between bacterial communities between any two pairs of groups (except for red macroalgae and brown macroalgae), suggesting that fungal communities were not as easily influenced by host phylogeny as bacterial communities (Table 1). The geographical differences may have played a role in the selection of microbial communities, with some association between their community structure and the location of sampling sites (Figure 5B). Fungal–algal interactions commence within the phycosphere with spore attachment and hyphal invasion, leading to colonization and the establishment of parasitic, mutualistic, endosymbiotic, or saprophytic fungi (Issa et al., 2014). Therefore, it is more difficult for fungi to than for bacteria attach to their hosts. In addition, fungi and bacteria reproduce differently, and fungi generally grow more slowly than bacteria, which may also result in relatively unstable fungal communities that are easily affected (Zheng et al., 2020). In the present study, it was hypothesized that due to the differences between fungi and bacteria, the fungi had weaker inter-relationships with their hosts than bacteria and preferred to free-swim in the seawater environment. Therefore, phylogenetic relationships affected fungi less than bacteria in the same environment, while fungi were more susceptible to environmental changes in different environments. Previous studies have also shown environmental differences in geographic location as the main factor influencing macroalgal β-diversity (Abdel-Gawad et al., 2014; Issa et al., 2014). Although the genotypes of macroalgae and seagrasses may only have a significant effect on bacterial community structure, they affect the α -diversity of both bacteria and fungi (Figure 3). In present study, the bacterial and fungal communities in seawater had higher Chao1 values, suggesting that macroalgae and seagrasses may recruit epiphytic microorganisms associated with themselves primarily from the seawater environment. In addition, the bacterial communities in seagrasses had a higher species richness and evenness than those in macroalgae. Unlike macroalgae, seagrasses are higher marine plants with roots, stems, and leaf tissues. Previous studies on terrestrial plants have shown that the sources of interleaf epiphytic microbiota include soil, seeds, and air (Gong and Xin, 2021). In the present study, it was speculated that although seagrasses and macroalgae are present in the same microbial pool (seawater), seagrasses may additionally acquire some bacteria associated with themselves from the soil and thus have a high species diversity.

### Variations in Biomarkers of Bacteria and Fungi

A non-parametric statistical test using “anosim” and “adonis” showed that, except for two groups (seagrass vs. brown macroalgae and seagrass vs. brown macroalgae) in fungal communities, the differences between groups were significantly greater than the differences within groups. These data indicated that the grouping of macroalgae and seagrasses in the present study is appropriate (Table 1). The LEfSe analysis showed that several taxa were candidate biomarkers for discriminating between the different groups. Differences in microbial recruitment by different types of hosts in the same environment were detected. For example, the brown algal cell wall has a unique chemical structure, consisting of a tight network of proteins and polysaccharides including fucoidan, which gives the cell wall a high degree of stability and accounts for 23% of its dry weight (Deniaud-Bouët et al., 2017). Fucoidin is degraded more slowly by the microbial community and is responsible for the sequestration of brown algal biomass (Arnosti, 2011; Deniaud-Bouët et al., 2014; Trevathan-Tackett et al., 2015). Recent studies have shown that the *Dokdonia*, *Reichenbachiella*, *C1-B045* and *Marinomonas* genera, as biomarkers, play an important role in the degradation of carrageenan (Park et al., 2018), polysaccharides (Wietz et al., 2015), oil hydrocarbons (Peng et al., 2020), and polymers (Delacuvellerie et al., 2021), which would explain why the degradation-related bacteria had a high abundance in brown macroalgae. Furthermore, biomarkers were detected in seagrasses. These included *Granulosicoccus*, which was reported to be isolated from seagrasses (Kurilenko et al., 2010), and *Lutimonas*, which was reported to have high content in sediments (Zhang et al., 2017). In the same way that seagrasses have a high alpha diversity, it can be speculated that seagrasses may recruit their own microorganisms from both seawater and sediment environments, which would account for their high number of biomarkers. In addition, the *NS5 marine group* and *SUP05 cluster* genera in seawater were strong indicators of eutrophication (Kopprio et al., 2021). The *NS5 marine group* represents one of the most ubiquitous flavobacterial bacterioplankton groups associated with marine blooms, and is generally associated with organic matter degradation (Signori et al., 2018). SUP05 *cluster* bacteria are sulfur-oxidizing bacteria broadly distributed in oxygen minimum zones that have the potential to consume ammonium to produce nitrite under anoxic conditions (Shah et al., 2017). These results reflect the potential functions of seawater microbial communities.

### Variations in Functional Groups of Bacteria and Fungi

The heatmap results showed that the functional groups of epiphytic bacteria in macroalgae and seagrasses were very different from those of epiphytic bacteria in seawater. The data support the findings of some previous studies, revealing that the bacterial community composition in macroalgae is driven mainly by functional genes rather than by taxonomic or phylogenetic composition (Egan et al., 2013; Roth-Schulze et al., 2016; Cirri and Pohnert, 2019; Selvarajan et al., 2019). With respect to C metabolism, xenobiotic biodegradation (hydrocarbon degradation, aromatic compound degradation, aliphatic non-methane hydrocarbon degradation, aromatic hydrocarbon degradation, ligninolysis, cellulolysis, and plastic degradation) and fermentation functional groups were higher in macroalgae and seagrasses than in seawater. These results suggest that macroalgae and seagrass epiphytes convert large molecules of organic matter to small molecules. The data support the findings of some previous studies that macroalgae and seagrasses are involved in the mineralization of dissolved organic matter in rocky intertidal oligotrophic environments (Selvarajan et al., 2019). Thus, macroalgae and seagrass epiphytes accelerate the C mineralization process and contribute significantly to the degradation of organic matter. With respect to N metabolism, the bacterial groups associated with nitrate reduction and ureolysis were also higher in macroalgae and seagrass groups than in seawater, which may imply that macroalgae and seagrasses play an important role in nitrogen mineralization. These results also reveal that some predicted animal and plant-related pathogenic functional groups are more abundant in seawater than in macroalgae and seagrasses. Previous studies have shown that seaweeds, lacking an elaborate *in situ* immune system, may exploit their symbiotic interactions with epiphytic bacteria by relying on the secondary metabolites of epiphytic bacteria as chemical defenses (Wahl et al., 2012). The results of the present study show that macroalgae and seagrasses may inhibit disease-causing bacterial groups of animals and plants.

FUNGuild predictions showed that undefined saprotroph was significantly more abundant in macroalgae and seagrasses than in seawater. Many studies have found that undefined saprotroph participate in organic matter degradation (Yan et al., 2018; Schmidt et al., 2019). These results suggest that the epiphytic fungi of macroalgae and seagrasses play as important a role in degradation as epiphytic bacteria. Although the FAPROTAX and FUNGuild databases provide information on the functional categories of microorganisms, they are only a tool to use with DNA sequencing results for functional prediction. Future studies on microbial function should use RNA-based work, such as meta-transcriptomic sequencing which would provide more strong evidence for microbial function.

## Conclusion

This study investigated the diversity, differences, and functions of the epiphytic microbiota of macroalgae and seagrasses in an intertidal zone. The results suggest that host phylogeny may play a role in influencing the structure of bacterial communities, and geographical differences may play a role in influencing the structure of fungal communities. The species richness in seagrasses was higher than that in macroalgae. The bacterial communities in the marine macrophytes were dominated by Proteobacteria and Bacteroidota, while the fungal communities were dominated by Ascomycota, Chytridiomycota, and Basidiomycota. The LEfSe analysis revealed a high abundance of degradation-associated bacteria in brown algae, which may be used to degrade fucoidin. Functional predictions showed that microbial communities on different host species were functionally distinct. Epiphytic microbes of macrophytes play an important role in geochemical cycling. In future work, it will be necessary to take into account the metabolites of the host and combine that information with the functional data to gain insights into epiphyte–host interactions.

## Data Availability Statement

The original contributions presented in the study are included in the article/Supplementary Material, further inquiries can be directed to the corresponding author/s.

## Author Contributions

JC conceived, designed the study, and wrote the manuscript. JC, YZ, TS, and ZY performed the experiments. TQ, SL, and MZ contributed to materials and analysis tools. XT and YW revised the manuscript. All authors have approved the final manuscript.

## Conflict of Interest

The authors declare that the research was conducted in the absence of any commercial or financial relationships that could be construed as a potential conflict of interest.

## Publisher’s Note

All claims expressed in this article are solely those of the authors and do not necessarily represent those of their affiliated organizations, or those of the publisher, the editors and the reviewers. Any product that may be evaluated in this article, or claim that may be made by its manufacturer, is not guaranteed or endorsed by the publisher.

## Supplementary Material

Supplementary Figure 1 Rarefaction curve analysis of all samples. **(A)** Rarefaction curves of the bacterial community. **(B)** Rarefaction curves of the fungal community.

Supplementary Figure 2 Relative abundance of bacteria and fungi per sample in this study. **(A)** Bacterial community composition at the phylum level. **(B)** Bacterial community composition at the genus level. **(C)** Fungal community composition at the phylum level. **(D)** Fungal community composition at the genus level.

Supplementary Figure 3 Differentially abundant taxa; the length of the histogram represents the impact of the different species. **(A)** Linear discriminate analysis (LDA = 3) of biomarker bacteria. **(B)** Linear discriminate analysis (LDA = 2) of biomarker fungi.

Supplementary Table 1 Microbial community sequencing data of the seawater, green macroalgae, red macroalgae, brown macroalgae, and seagrasses.

Supplementary Table 2 A comparison of community alpha diversities between seawater, green macroalgae, red macroalgae, brown macroalgae, and seagrasses.
