# Supplementary figures and images for: Composition and Functional Diversity of Epiphytic Bacterial and Fungal Communities on Marine Macrophytes in an Intertidal Zone
Source: Front Microbiol. 2022 Mar 18;13:839465. doi: 10.3389/fmicb.2022.839465 (PMC8972133; doi:10.3389/fmicb.2022.839465)

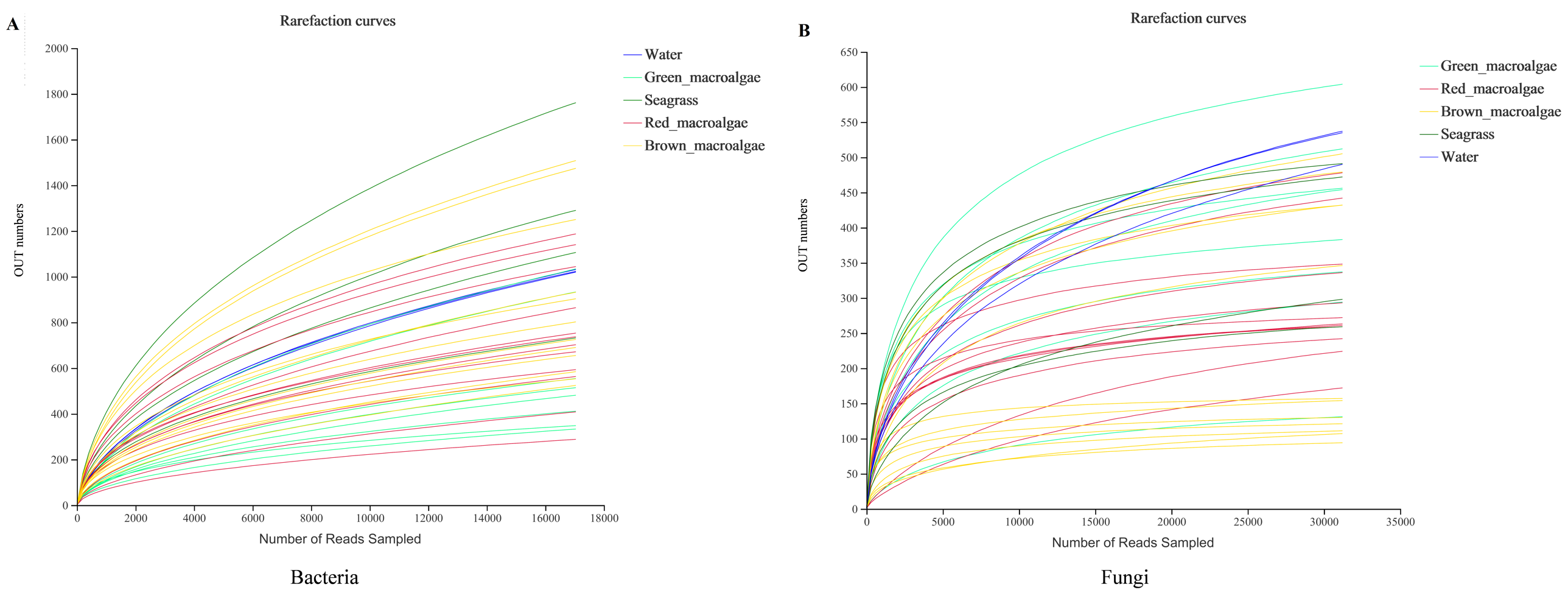

Supplement: Supplementary Figure 1 — Rarefaction curve analysis of all samples. (A) Rarefaction curves of the bacterial community. (B) Rarefaction curves of the fungal community. [file Image_1.TIF]

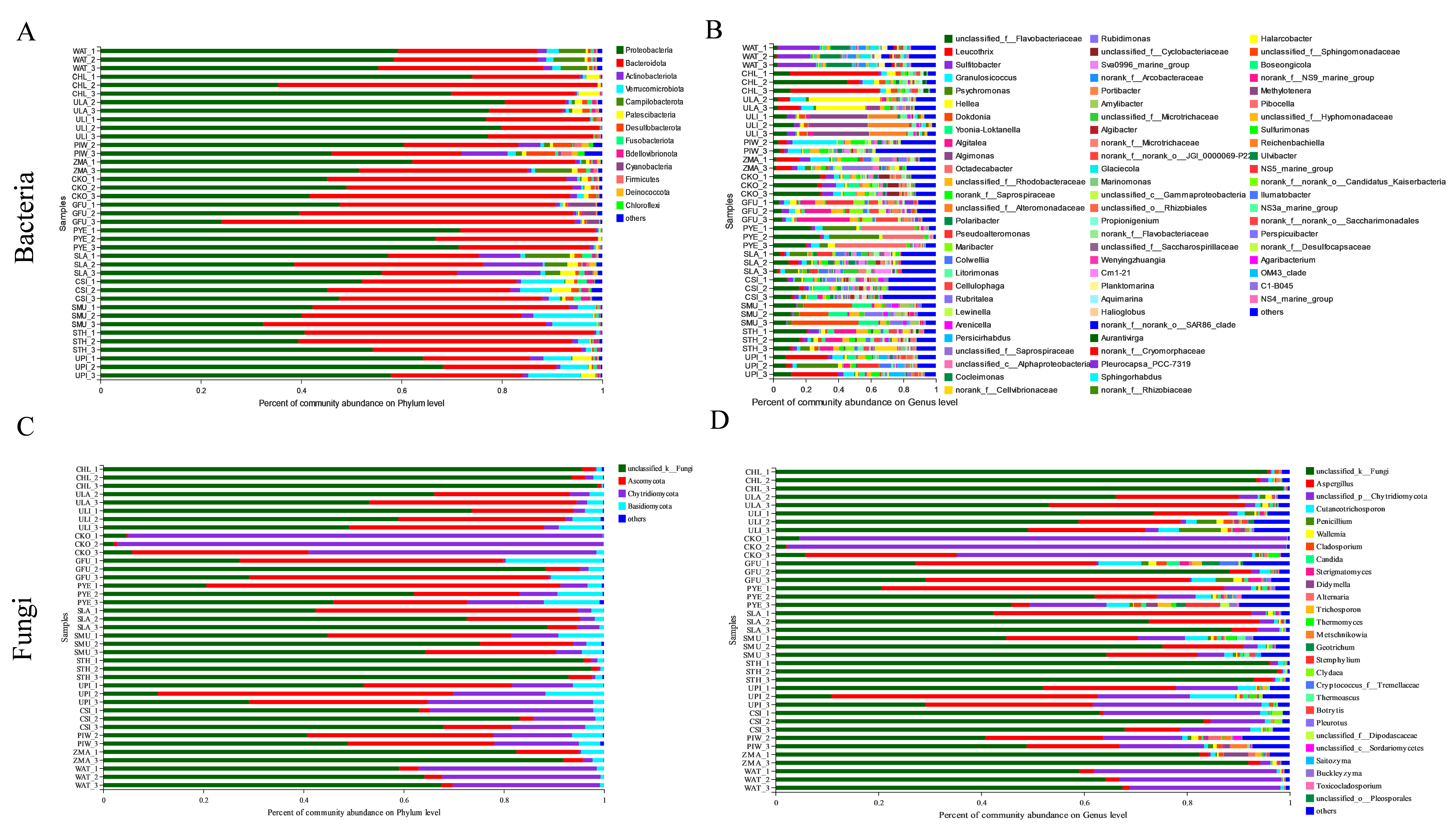

Supplement: Supplementary Figure 2 — Relative abundance of bacteria and fungi per sample in this study. (A) Bacterial community composition at the phylum level. (B) Bacterial community composition at the genus level. (C) Fungal community composition at the phylum level. (D) Fungal community composition at the genus level. [file Image_2.TIF]

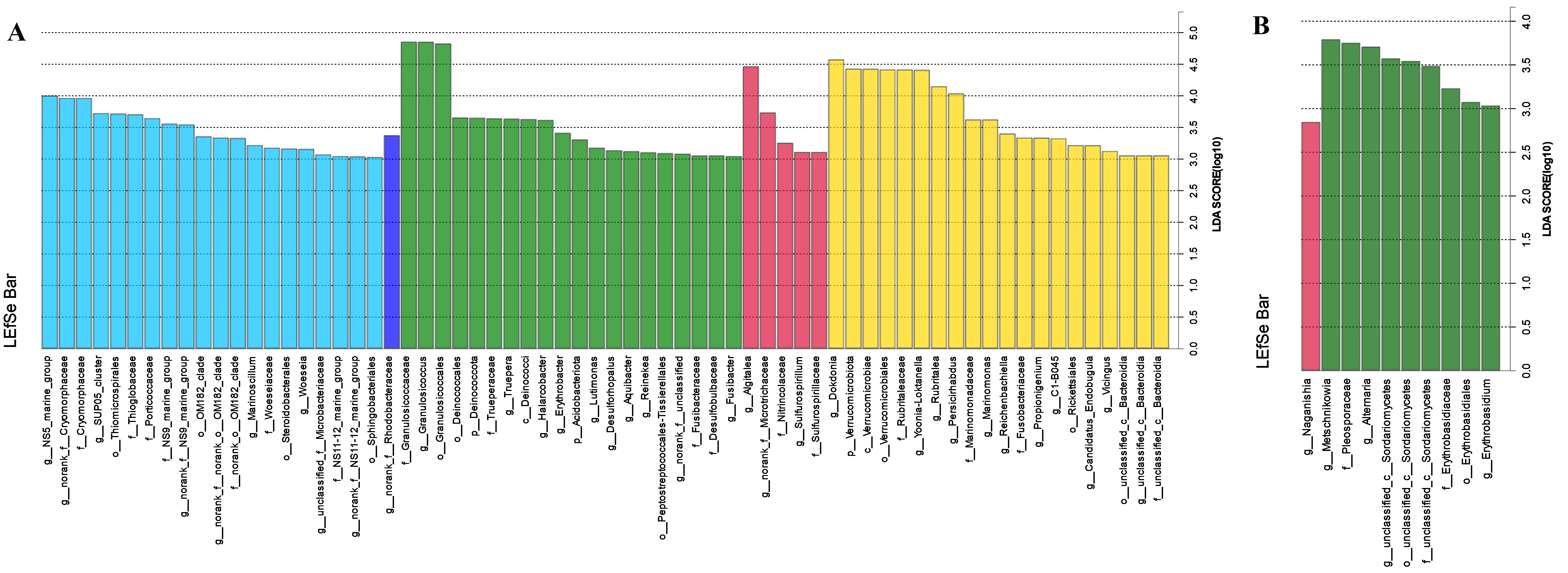

Supplement: Supplementary Figure 3 — Differentially abundant taxa; the length of the histogram represents the impact of the different species. (A) Linear discriminate analysis (LDA = 3) of biomarker bacteria. (B) Linear discriminate analysis (LDA = 2) of biomarker fungi. [file Image_3.TIF]
